# The influence of item order of the Household Food Security Survey Module on the assessment of food insecurity in households with children

**DOI:** 10.1017/S1368980022001239

**Published:** 2022-09

**Authors:** Isabel Maia, Milton Severo, Carla Lopes, Ana Cristina Santos

**Affiliations:** 1 EPIUnit – Instituto de Saúde Pública, Universidade do Porto, 4050-600 Porto, Portugal; 2 Laboratório para a Investigação Integrativa e Translacional em Saúde Populacional (ITR), Porto, Portugal; 3 Departamento de Ciências da Saúde Pública e Forenses e Educação Médica, Faculdade de Medicina, Universidade do Porto, Porto, Portugal

**Keywords:** Food insecurity, Item order, Households with children, Generation XXI, National surveys, Portugal

## Abstract

**Objective:**

Changes in the item order of the US Household Food Security Survey Module (USHFSSM) were performed throughout time. This study aimed to compare the psychometric properties of the general and specific factors of the 2000 and 2012 versions of the USHFSSM to measure the construct of food insecurity in two Portuguese samples of households with children.

**Design:**

Cross-sectional.

**Setting:**

Portugal.

**Participants:**

An adaptation of the 2000 version was applied to 839 adults (from households with children aged 7–17 years) from the National Food, Nutrition and Physical Activity Survey 2015–2016, while the 2012 version was used among 2855 families from the Generation XXI birth cohort.

**Results:**

The 2000 version showed to have a stronger ωh than the 2012 version (0·89 *v*. 0·78 for the general factor), as well as eigenvalues higher than 1 for the general factor (eigenvalues equal to 9·54, 0·97 and 0·80, for the general factor, specific factor 1 and specific factor 2, respectively), while the 2012 version had also the contribution of specific factors to explain food insecurity (eigenvalues equal to 9·40, 2·40 and 1·20, for general factor and specific factors 1 and 2, respectively). Good internal consistency (ωt = 0·99, for both versions) was obtained.

**Conclusions:**

In conclusion, the 2000 and 2012 versions of the USHFSSM showed good psychometric properties; however, the 2000 version has stronger general factor, while the 2012 version also has the contribution of specific factors.

Food insecurity is an important public health issue of societies^(1–3)^. Going back to the 1980s, attention was given to food insecurity and hunger in the USA and became imperative the need for the development of tools to measure this phenomenon^(4)^.

Throughout time, different instruments for the food security status assessment have been developed. Among the existing instruments, the US Household Food Security Survey Module (USHFSSM) of the Department of Agriculture – developed with the contribution of the Radimer/Cornell Food Security Scale and the Community Childhood Hunger Identification Project^(5–7)^ – has been widely used, subject to cultural adaptations and used in different socio-economic and cultural contexts^(8)^. This instrument showed to be a valid and reliable scale^(9–11)^ and is composed by eighteen items (three items related to the household, seven related to the adults and eight items answered if there are children within the household)^(12,13)^. The guidelines to measure food security status were reported in the *Guide to Measuring Household Food Security*, of 2000^(13)^.

Although the content of the eighteen items of the USHFSSM remained essentially the same, compared to the version reported in 2000, some changes in the scale were performed along the years^(10)^. These changes, recommended by the Committee on National Statistics of the National Academies^(10,14)^, are concerned about the reorder of the items. Also, minor changes in some items to standardise the wording of resources constraint were applied. Thus, all child-referenced items were grouped after the household- and adult-referenced items of the USHFSSM^(10,14)^.

Although the aforementioned changes in the USHFSSM were not subject to confirmatory research^(14)^, according to the literature, changes in the sequence/position of the items can affect the individuals’ responses^(15)^ and the factor structure^(16)^.

An adaptation of the version reported in 2000^(13,17)^, as well as the most recent version of the USHFSSM of 2012^(12)^, was used in the assessment of food security status in two Portuguese samples. Thus, it is of utmost relevance to understand if the performance of the two different versions in measuring the construct of food insecurity is the same.

Therefore, this study aimed to compare the psychometric properties of the general and specific factors of the 2000 and 2012 versions of the USHFSSM measuring the food insecurity construct in households with children among two Portuguese population-based samples.

## Methods

### Study design and participants

The present study was based on data from the National Food, Nutrition and Physical Activity Survey (IAN-AF 2015–2016)^(17,18)^ and from the 10-year-old follow-up evaluation of the Portuguese population-based birth cohort, Generation XXI (G21) (2015–2017)^(19,20)^.

The IAN-AF was conducted to collect nationwide and regional information on dietary habits, physical activity and their relation with health determinants. In brief, a representative sample of the Portuguese population, aged between 3 months and 84 years, was selected by multistage sampling, using the Primary Health Care Units as sampling frame. IAN-AF procedures and selection methods are described elsewhere^(17,18)^. Data were collected from October 2015 to September 2016, and two interviews (8 to 15 d apart) were carried out by computer-assisted personal interviewing. Food security status data were collected, in the second interview. A total of 6553 individuals attended the first interview; of those, 5819 completed the two moments of evaluation^(21)^. For the current study, data on IAN-AF adult participants living in households with children aged between 7 and 17 years of age were considered (*n* 920). Questionnaires with missing data on at least one item of the food insecurity scale were excluded from the analysis (*n* 81), comprising a final sample of 839 participants.

The G21 birth cohort enrolled 8647 live newborns born in the five public maternity units of Porto Metropolitan Area, Portugal, between April 2005 and August 2006^(19,20)^. Follow-up evaluations of the cohort took place at 4, 7 and 10 years of age. The 10-year-old follow-up evaluation occurred between July 2015 and July 2017, and 6397 children were assessed. As part of this follow-up evaluation, a subsample of children was consecutively invited to food security status assessment and 2942 children’s families were evaluated. Singletons or one of the children, in the case of multiple births or siblings, were considered for analysis (*n* 2867). Similar to the IAN-AF, the questionnaires with missing information on at least one item of the module were excluded from the analysis (*n* 12), leading to a final sample of 2855 participants.

### Data collection

Data on food security status were collected by applying two versions of the USHFSSM, whereby individuals are inquired if they were capable of to afford the food they need, in the previous 12 months^(12,13)^.

In IAN-AF, data on household food security status were collected by trained researchers, through a computer-assisted personal interview, using a slightly modified version of the questionnaire reported by Bickel et al.^(13)^, in 2000 (see online supplementary material, Supplemental Table 1), as previously described^(13,17)^. The IAN-AF questionnaire on food security status, which was adapted for Portugal by the Instituto Nacional de Saúde Dr. Ricardo Jorge and the Economic Research Service of the US Department of Agriculture, was answered by an adult (18 years of age or more) of the household. From now on, to refer to this questionnaire, the denomination ‘2000 version’ of the USHFSSM will be used throughout the present study.

In G21, the household food security status was assessed through the USHFSSM (2012 version)^(12)^ (see online supplementary material, Supplemental Table 1), which was applied through face-to-face interview, to children’s parent/accompanying adult, as long as he/she belonged to the children’s household and was 18 years of age or more. The USHFSSM was translated from English to Portuguese by two researchers with expertise in nutritional sciences and epidemiology and afterwards back-translated into English by an independent professional translator and concomitantly native English speaker (blinded from the original version). The two versions were compared to guarantee equivalence, and no relevant differences were verified. In the items ‘*(I/we) couldn’t afford to eat balanced meals*’ and ‘*(I/We) couldn’t feed (my/our) child/the children a balanced meal, because (I/we) couldn’t afford that*’, the term *balanced meal(s)* was substituted by *complete, healthy meal(s)* (refeição(ões) completa(s) e saudável(eis), in Portuguese) due to the need of cultural adaptation. In the same way as IAN-AF, to refer to this questionnaire, the wording ‘2012 version’ of the USHFSSM will be used along with this study.

In addition to the data on food security status, in the IAN-AF, data on the age (in years) of the participant and education level – classified into none or first cycle of basic education (≤ 4 years), second cycle of basic education (6 years), third cycle of basic education (9 years), secondary education (12 years) and higher education (> 12 years) – were collected. Information on the average household monthly income, categorised into lower than or equal to 970 €, 971 € to 1455 €, 1456 € to 1940 € and higher than 1940 €, and on household size (as the number of persons living within the household), was taken. Also, considering the categories of the data on the number of household members according to age – < 7 years, 7–17 years, 18–64 years and ≥ 65 years – the number of all children within the household and those aged 7–17 years were retrieved.

In the G21 birth cohort, as the majority of respondents (79·5 %) to the USHFSSM was the child’s mother, data on maternal age and education were used. To enable the comparison with IAN-AF categories, maternal education was collected as completed years of schooling but was then categorised into none or first cycle of basic education (≤ 4 years), second cycle of basic education (5–6 years), third cycle of basic education (7–9 years), secondary education (10–12 years) and higher education (> 12 years).

Also, information on household income, categorised into lower than or equal to 1000 €, 1001 € to 1500 €, 1501 € to 2000 € and higher than 2000 €, and household size, as the number of persons living within the household, was obtained. The number of all children within the household was also considered, as well as those aged 7–17 years, to be similar to IAN-AF categories.

### Statistical analysis

The two versions of the scale were compared and for both versions, original items of the scale were used for the analysis. Items that were not endorsed (floor effect) were not accounted for in the analysis. The USHFSSM has items related to cutting the size of meals, skipping meals and not eating for a whole day (occurrence (‘yes’ or ‘no’) and frequency (‘almost every month’, ‘some months but not every month’ or ‘only 1 or 2 months’)). For the analysis, the items related to the frequency of cutting the size of meals, skipping meals and not eating for a whole day were not accounted for because they presented a similar proportion of endorsement to the items asking the occurrence (‘yes’ or ‘no’).

Firstly, factor analysis was used to test the configural invariance (if the same factor structure)^(22,23)^ between the two versions of the USHFSSM. Thus, exploratory factor analysis was conducted applying hierarchical factor analysis to assess the general factor saturation of both versions. Factor analysis with oblique rotation, followed by Schmid–Leiman transformation,^(24)^ was applied to determine the general factor and the specific factors, in both versions. Also, in the hierarchical factor analysis, the tetrachoric correlation was applied as the items were dichotomous. We only showed the values of factors loading higher than 0·2^(25)^.

To estimate separately the general factor saturation from the internal consistency, coefficients omega hierarchical (ωh) and omega total (ωt) of McDonald^(26)^ were calculated.

As a second analysis, we also did 1-factor 2-parameter logistic (2-PL) item response theory models for both versions of the USHFSSM. The item response theory models describe the relationship between a latent trait, item properties and answers to the items^(27)^. In 1-factor 2-PL model, individuals’ responses to a binary item are influenced by the latent trait level, the item difficulty, as well as the item discrimination (or standardised factor loading, to quantify the discrimination)^(28)^. A higher value of difficulty represents a lower probability of providing an affirmative response to an item^(27)^. The higher the discrimination (or standardised factor loading), the higher the correlation of the item with the latent trait and higher is the capacity of the item to differentiate individuals with high *v*. low trait levels^(28)^.

We tested three 1-factor 2-PL models, including all the items included in the analysis, the household/adult-referenced items and the child-referenced items.

To assess the goodness of fit of original data to this particular model, two-way marginal was checked and parametric bootstrap re-samples were drawn with a total of 199 replicas, refitting the 1-factor 2-PL item response theory models to these re-samples. Chi-square statistic and likelihood ratio test were calculated and compared with the original statistics. Item correlations are presented in the Supplemental Table 2, and item-fit chi-square statistics are presented in Supplemental Table 3.

To characterise the IAN-AF and G21 samples, continuous variables were described as mean and standard deviation (sd) and compared using Student’s *t* test. Categorical variables were described as absolute and relative frequencies and compared using the chi-square test. Effect size measures were also calculated (Cohen’s d and Cramer’s V).

For the two versions of the USHFSSM, the household’s raw scores of both versions, as the sum of affirmative responses of each item of those included in the analyses, were calculated. To assess if the score is more related to child or household characteristics, the relationship between socio-demographic characteristics and the household’s raw score (of all the items and the child-referenced items included in the analysis) of the versions was explored. For that purpose, data on the respondent’s age, education, household income and household size, as well as the number of all children and those aged 7–17 years in the household, were used. As the household’s raw score showed a skewed distribution, a log transformation to the household’s raw score plus 1 was applied. In this analysis, continuous variables were presented using geometric mean and geometric standard deviation (GSD). The household’s raw scores of both versions were correlated with continuous variables using the Pearson correlation coefficients and compared across categorical variables using the Student’s *t* test and ANOVA, as appropriate.

To compare the effect size of the relationship between the household’s raw scores (of all the items and the child-referenced items) and the socio-demographic characteristics in both versions, the partial eta squared and Cohen’s d, as appropriate, were used. Values of partial eta squared of 0·01, 0·06 and 0·14 and Cohen’s d of 0·2, 0·5 and 0·8 were considered small, medium and large effects, respectively^(29,30)^.

R software (The R Project for Statistical Computing), version 3.4.0 for Windows, was used to estimate hierarchical factor and the omega coefficients, using psych package^(31)^. Also, IBM SPSS (Statistical Package for Social Sciences), version 25.0, was used. A significance level of 0·05 was considered.

## Results

The characteristics of the IAN-AF and G21 participants are presented in Table 1. In both samples, a similar respondent and maternal mean age were observed (mean (sd) = 41·4 (10·3) and mean (sd) = 41·4 (5·0) years, for IAN-AF and G21, respectively (*P* = 0·949)). Compared to the G21, the IAN-AF included a higher proportion of less-educated individuals (11·6 % *v*. 4·8 %; *P* < 0·001), low-income households (31·9 % *v*. 24·6 %; *P* < 0·001) and a lower proportion of households with two or more children aged 7 to 17 years (26·2 % *v*. 37·4 %; *P* < 0·001) (Table 1).

**Table 1:** Characterisation of the samples

	2000 version (*n* 839)		2010 version (*n* 2855)			
	*n*	%	*n*	%	*P*-value	Effect size‡
Age*						
Mean	41·4		41·4		0·949	0·004
sd	10·3		5·0			
Education*,†					<0·001	0·119
≤ 4 years	97	11·6	138	4·8		
6 years | 5–6 years	98	11·7	380	13·4		
9 years | 7–9 years	171	20·4	580	20·4		
12 years | 10–12 years	244	29·1	833	29·3		
> 12 years	228	27·2	912	32·1		
Missing (*n*)	1		12			
Household monthly income†					<0·001	0·073
≤ 970 € | ≤ 1000 €	250	31·9	683	24·6		
971–1455 € | 1001–1500 €	198	25·3	817	29·4		
1456–1940 € | 1501–2000 €	131	16·7	542	19·5		
> 1940 € | > 2000 €	204	26·0	740	26·6		
Missing (*n*)	56		73			
Household size†					0·629	0·022
2 persons	44	5·2	124	4·3		
3 persons	240	28·6	861	30·2		
4 persons	410	48·9	1377	48·3		
≥ 5 persons	145	17·3	491	17·2		
Missing (*n*)	0		2			
Number of children in the household					<0·001	0·124
1 child	481	57·3	1216	42·6		
2 children	308	36·7	1398	49·0		
≥ 3 children	50	6·0	239	8·4		
Missing (*n*)	0		2			
Number of children aged 7 to 17 years in the household					<0·001	0·099
1 child	619	73·8	1785	62·6		
≥ 2 children	220	26·2	1068	37·4		
Missing (*n*)	0		2			

* Regarding maternal information in the Generation XXI birth cohort, as 79·5 % of the respondents to the US Household Food Security Survey Module was the child’s mother.

† *n* (%).

‡ Cohen’s d and Cramer’s V were used for continuous and categorical variables, respectively.

In both versions of the scale, the most frequently endorsed item was related to worrying that food would run out before (the family) got money to buy more (18·4 % and 18·7 %, respectively, in 2000 and 2012 versions). Items related to relying on ‘*only a few kinds of low-cost food to feed children*’, ‘*food bought just did not last*’ and ‘*couldn’t afford to eat balanced meals*’ (either adults or children) were also items frequently endorsed in both 2000 and 2012 versions (Table 2). The less endorsed item corresponded to the most severe one, related to children not eating for a whole day (0·0 % and 0·04 % in 2000 and 2012 versions).

**Table 2:** Factor analysis, using tetrachoric correlations, with Schmid–Leiman factor loadings rotation of the 2000 and 2012 versions of the US Household Food Security Survey Module

	2000					2012				
Items*	*n*	%†	g	F1	F2	*n*	%†	g	F1	F2
*‘(I/We) worried whether (my/our) food would run out before (I/we) got money to buy more’. Was that often true, sometimes true, or never true for (you/your household) in the last 12 months?*	154	18·4	0·80		0·36	533	18·7	0·68	0·64	
*‘The food that (I/we) bought just didn’t last, and (I/we) didn’t have money to get more’. Was that often, sometimes, or never true for (you/your household) in the last 12 months?*	102	12·2	0·85		0·35	185	6·5	0·77	0·56	
*‘(I/we) couldn’t afford to eat balanced meals’. Was that often, sometimes, or never true for (you/your household) in the last 12 months?*	135	16·1	0·85		0·31	140	4·9	0·76	0·47	
*In the last 12 months, since last (name of current month), did (you/you or other adults in your household) ever cut the size of your meals or skip meals because there wasn’t enough money for food?*	20	2·4	0·88	0·38		48	1·7	0·82	0·42	
*How often did this happen?*	17	2·0	–	–	–	33	1·2	–	–	–
*In the last 12 months, did you ever eat less than you felt you should because there wasn’t enough money for food?*	28	3·3	0·85	0·30		50	1·7	0·83	0·45	
*In the last 12 months, were you every hungry but didn’t eat because there wasn’t enough money for food?*	14	1·7	0·92	0·33		21	0·7	0·85	0·36	0·24
*In the last 12 months, did you lose weight because there wasn’t enough money for food?*	9	1·1	0·88	0·47		18	0·6	0·86	0·36	0·24
*In the last 12 months, did (you/you or other adults in your household) ever not eat for a whole day because there wasn’t enough money for food?*	4	0·5	0·80	0·42		2	0·1	0·66	0·22	0·24
*How often did this happen?*	4	0·5	–	–	–	2	0·1	–	–	–
*‘(I/we) relied on only a few kinds of low-cost food to feed (my/our) child/the children) because (I was/we were) running out of money to buy food’. Was that often, sometimes, or never true for (you/your household) in the last 12 months?*	127	15·1	0·86		0·43	253	8·9	0·73	0·52	
*‘(I/We) couldn’t feed (my/our) child/the children) a balanced meal, because (I/we) couldn’t afford that’. Was that often, sometimes, or never true for (you/your household) in the last 12 months?*	107	13·8	0·88		0·42	71	2·5	0·81	0·45	
*‘(My/Our child was/The children were) not eating enough because (I/we) just couldn’t afford enough food’. Was that often, sometimes, or never true for (you/your household) in the last 12 months?*	19	2·3	0·90	0·25		31	1·1	0·82	0·39	
*In the last 12 months, since (current month) of last year, did you ever cut the size of (your child’s/any of the children’s) meals because there wasn’t enough money for food?*	7	0·8	0·81	0·22		19	0·7	0·84	0·31	0·28
*In the last 12 months, did (child’s name/any of the children) ever skip meals because there wasn’t enough money for food?*						4	0·1	0·84		0·48
*How often did this happen?*	7	0·8	–	–	–	4	0·1	–	–	–
*In the last 12 months, (was your child/were the children) ever hungry but you just couldn’t afford more food?*	6	0·7	0·82	0·34		3	0·1	0·85		0·52
*In the last 12 months, did (your child/any of the children) ever not eat for a whole day because there wasn’t enough money for food?*	0	0·0	–	–	–	1	0·04	0·73		0·55
Eigenvalues			9·54	0·97	0·80			9·40	2·40	1·20
Explained variance (%)			73·38	7·46	6·15			62·67	16·00	8·00
Omega total			0·99	0·98	0·96			0·99	0·97	0·92
Omega hierarchical			0·89	0·85	0·80			0·78	0·74	0·69

F1, specific factor 1; F2, specific factor 2; g, general factor.

* Items wording of the 2012 version of the US Household Food Security Survey Module.

† *n* (%) of affirmative responses in each of the items. Responses as yes, often, sometimes, almost every month and some months but not every month are considered affirmative.

All the items, in both versions of the USHFSSM, presented factor loadings higher than 0·3 for the general factor. The majority of factor loadings of the specific factors were higher in 2012 than in the 2000 version (Table 2), and both versions of the USHFSSM revealed good internal consistency (ωt = 0·99, for both versions) (Table 2).

The 2000 version showed to have stronger ωh than the 2012 version (0·89 *v*. 0·78 for the general factor) and eigenvalues higher than 1 for the general factor (eigenvalues equal to 9·54, 0·97 and 0·80, for the general factor, specific factor 1 and specific factor 2, respectively), meaning a higher contribution of the general factor in explaining food insecurity. On the other hand, the 2012 version presented eigenvalues higher than 1 for both general (eigenvalue equal to 9·40) and specific factors (eigenvalues equal to 2·40 and 1·20, for specific factors 1 and 2, respectively), representing also a high contribution of the specific factors in explaining the construct under evaluation (Table 2).

These results were confirmed by the item response theory models applied to both versions of the USHFSSM (see online supplementary material, Supplemental Table 4).

The geometric mean raw score (GSD) was higher in the IAN-AF than in G21 sample, using the 2000 and 2012 versions, respectively. For both scale versions, significantly higher household’s raw scores (for all item and child-referenced items) were observed among lower educated respondents and in low-income households. No differences regarding the age of the respondents were observed (Table 3).

**Table 3:** Raw score of the 2000 and 2012 versions of the US Household Food Security Survey Module, according to the participants’ characteristics of National Food, Nutrition and Physical Activity Survey and Generation XXI birth cohort

	2000 (*n* 839)								2012 (*n* 2855)							
	All items				Only child-referenced items				All items				Only child-referenced items			
Raw score	0·41				0·18				0·25				0·08			
GM	0·90				0·49				0·62				0·30			
GSD	*r* §		*P*-value		*r* §		*P*-value		*r* §		*P*-value		*r* §		*P*-value	
Age (years)	0·043		0·209		0·046		0·180		−0·037		0·051		−0·009		0·637	
	GM	GSD	*P*-value	η^2^ ||	GM	GSD	*P*-value	η^2^ ||	GM	GSD	*P*-value	η^2^ ||	GM	GSD	*P*-value	η^2^ ||
Education*			<0·001	0·141			<0·001	0·131			<0·001	0·101			<0·001	0·063
≤ 4 years	1·28	1·44			0·60	0·82			0·82	1·12			0·31	0·67		
6 years† | 5–6 years‡	0·85	1·18			0·43	0·65			0·48	0·79			0·15	0·40		
9 years† | 7–9 years‡	0·50	0·95			0·19	0·47			0·44	0·81			0·14	0·38		
12 years† | 10–12 years‡	0·29	0·69			0·11	0·36			0·18	0·48			0·04	0·20		
> 12 years	0·08	0·34			0·03	0·20			0·07	0·30			0·02	0·13		
Household monthly income			<0·001	0·175			<0·001	0·124			<0·001	0·128			<0·001	0·080
≤ 970 €† | ≤ 1000 €‡	1·02	1·26			0·42	0·69			0·64	0·94			0·22	0·50		
971–1455 €† | 1001–1500 €‡	0·38	0·83			0·19	0·47			0·26	0·58			0·06	0·24		
1456–1940 €† | 1501–2000 €‡	0·17	0·51			0·07	0·31			0·12	0·38			0·03	0·16		
> 1940 €† | > 2000 €‡	0·04	0·20			0·01	0·07			0·04	0·24			0·01	0·12		
Household size			<0·001	0·030			<0·001	0·020			<0·001	0·015			<0·001	0·014
2 persons	0·85	1·07			0·25	0·59			0·25	0·62			0·07	0·28		
3 persons	0·39	0·90			0·18	0·47			0·24	0·60			0·07	0·27		
4 persons	0·30	0·75			0·14	0·41			0·20	0·54			0·06	0·26		
≥ 5 persons	0·68	1·17			0·33	0·66			0·42	0·83			0·16	0·43		
Number of children in the household			0·008	0·011			0·003	0·014			<0·001	0·010			<0·001	0·009
1 child	0·38	0·86			0·16	0·46			0·25	0·61			0·07	0·29		
2 children	0·40	0·88			0·20	0·49			0·22	0·57			0·07	0·27		
≥ 3 children	0·85	0·28			0·42	0·70			0·46	0·88			0·17	0·46		
	GM	GSD	*P*-value	d	GM	GSD	*P*-value	d	GM	GSD	*P*-value	d	GM	GSD	*P*-value	d
Number of children aged 7 to 17 years in the household			0·455	0·059			0·088	0·144			0·175	0·054			0·027	0·089
1 child	0·40	0·86			0·17	0·46			0·24	0·60			0·07	0·28		
≥ 2 children	0·45	1·00			0·24	0·55			0·27	0·66			0·10	0·33		

d, Cohen’s d; GM, geometric mean; GSD, geometric standard deviation.

* Regarding maternal information in the Generation XXI birth cohort, as 79·5 % of the respondents to the US Household Food Security Survey Module was the child’s mother.

† National Food, Nutrition and Physical Activity Survey sample.

‡ Generation XXI sample.

§ Pearson’s correlation coefficient.

|| Partial eta squared.

In general, the effect size of the relationship between the socio-demographic characteristic and the household’s raw score was higher in 2000 than in the 2012 version.

## Discussion

In this study and using data from two population-based samples, we compared the psychometric properties of the general and specific factors of two versions – 2000 and 2012 – of the USHFSSM to measure the construct of food insecurity.

Firstly, we explored the factor structure of both scales. The main difference between the two versions was mainly related to the order of the items; in the most recent version, all child-referenced items were grouped at the end of the module^(10,14)^. Thus, and using the hierarchical factor analysis with Schmid–Leiman transformation, we analysed the structure of both versions of the USHFSSM, particularly, the existence of a general factor or specific factors regarding the construct of food insecurity, and according to our results, the factor structure showed not to be the same in the two versions. Food security status showed to be mainly explained by the general factor in the 2000 version, while in the 2012 version, the contribution of specific factors – one more related to household/adults-referenced items and another to child-referenced items (particularly the most severe items), was observed.

Concerning the factor structure, although both versions had a good contribution of the general factor in explaining the variance, the most recent version allowed a better distinction between the food insecurity among household/adults and children. This was also supported by the higher proportion of variance explained by the general factor in the 2000 version, compared to the 2012 version (73·4 % *v*. 62·7 %). The factor loadings of the specific factors in the 2012 version were higher than in the 2000 version, which corroborates the relevance of the specific factors for the construct of food insecurity. Supporting these findings, the 2012 version of the USHFSSM presented eigenvalues higher than 1^(32)^ in both general and specific factors, sustaining that the variance in food security status was also explained by the specific factors.

Although both versions of the USHFSSM had high ωh values, which corroborated the unidimensionality^(33)^ and higher variance explained by the general factor, the 2000 version had a higher ωh than the 2012 version.

According to a recent report, one of the statistical bias of the USHFSSM is that it represents, in fact, two latent traits – the severity of food insecurity among adults and food insecurity in children^(34,35)^. This is opposed to what is assumed using the Rasch model – food insecurity measured as a continuum, in which only one latent trait was measured^(34–36)^. Because of the existence of two latent characteristics, alternative classification has been explored, separating food insecurity among adults and children^(34)^, but there is no strong evidence in favour of that classification. Our results are in accordance with what has been assumed, that the scale reflects a single construct of food insecurity, supported by the high value of ωh observed.

Regarding the internal consistency and although Cronbach’s α is frequently used for its assessment, the report of other estimates of internal consistency has been recommended^(37)^. Thus, in our study, we used ωt to measure internal consistency, and both versions presented an excellent internal consistency, as values higher than 0·7 were observed^(38)^.

Using item response theory models, it was verified that all items are important to the construct and allowed the discrimination of individuals.

Although the scales are not comparable, both versions of the USHFSSM showed to be related to the socio-demographic characteristics of the samples, and the effect size of the differences was slightly higher for the 2000 version. The explored socio-demographic characteristics were mainly related to the household, and as in the 2000 version food insecurity is mainly explained by the general factor, this could justify the higher effect size observed in that sample.

Some limitations of the present study ought to be discussed. As food insecurity is a sensitive issue and as it has been collected based on individuals’ self-report, the possibility of social desirability bias and underestimation of the real condition of the household cannot be discarded. Furthermore, differences between the two samples regarding education, household monthly income, number of all children and those aged 7–17 years in the household were observed; however, the effect sizes were small. Also, the floor effect has to be mentioned, as a higher proportion of zero affirmative responses was observed for both versions (74·3 % and 78·8 %, for 2000 and 2012, respectively). Moreover, the two versions did not include exactly the same items, because the IAN-AF version had differences compared to the original 2000 version^(13)^. The major differences were related to the items regarding children within the household skipping meals and children cutting the size of meals that were combined into a single item in the version used in IAN-AF. However, this item had a low proportion of endorsement and thus probably did not largely influence the results. Also, the possibility of underestimation of the number of children within the households in G21 sample cannot be discarded.

In G21, beyond parents, step-parents, grandparents, stepsiblings or siblings, data on the number of other (family) members were collected. But the age of those members was not, and some families may account with children as those members. However, we believe that this number may be limited.

Nevertheless, this study was strengthened by the use of data from two population-based Portuguese samples, with similar characteristics. Considering the relevance of having a food insecurity measure for both monitoring and research, this study provided relevant insights into the psychometric properties of two versions of the USHFSSM^(12)^ among Portuguese households with children.

In conclusion, the 2000 and 2012 versions of the USHFSSM showed good psychometric properties; however, the 2000 version has stronger general factor, while the 2012 version also has the contribution of specific factors. Therefore, the scales are not comparable considering that they did not have the same factor structure. Nevertheless, the associations between the raw score and the socio-demographic characteristics were similar in both versions.

## Supplementary material

For supplementary material accompanying this paper visit https://doi.org/10.1017/S1368980022001239. click here to view supplementary material
