# Supplementary material for: The influence of item order of the Household Food Security Survey Module on the assessment of food insecurity in households with children
Source: Public Health Nutr. 2022 May 23;25(9):2371–9. doi: 10.1017/S1368980022001239 (PMC9991806; doi:10.1017/S1368980022001239)
Supplement: Supplementary file 1 [file S1368980022001239sup001.docx]

| **Supplementary Table 1:** Original 2000 and 2012 versions of the United States Household Food Security Survey Module | | | | | |
| --- | --- | --- | --- | --- | --- |
| **2000 version** | |  | **2012 version** | | **Item correspondence (2000 to 2012)** |
|  | *Now I’m going to read you several statements that people have made about their food situation. For these statements, please tell me whether the statement was often true, sometimes true, or never true for (you/your household) in the last 12 months, that is, since last (name of current month).* |  |  | *Now I’m going to read you several statements that people have made about their food situation. For these statements, please tell me whether the statement was often true, sometimes true, or never true for (you/your household) in the last 12 months—that is, since last (name of current month).* |  |
| 2 | The first statement is “(I/We) worried whether (my/our) food would run out before (I/we) got money to buy more.” Was that often true, sometimes true, or never true for (you/your household) in the last 12 months? [ ] Often true [ ] Sometimes true [ ] Never true [ ] DK or Refused |  | HH2 | The first statement is “(I/We) worried whether (my/our) food would run out before (I/we) got money to buy more.” Was that often true, sometimes true, or never true for (you/your household) in the last 12 months? [ ] Often true [ ] Sometimes true [ ] Never true [ ] DK or Refused | 2 - HH2 |
| 3 | “The food that (I/we) bought just didn’t last, and (I/we) didn’t have money to get more.” Was that often, sometimes, or never true for (you/your household) in the last 12 months? [ ] Often true [ ] Sometimes true [ ] Never true [ ] DK or Refused |  | HH3 | “The food that (I/we) bought just didn’t last, and (I/we) didn’t have money to get more.” Was that often, sometimes, or never true for (you/your household) in the last 12 months? [ ] Often true [ ] Sometimes true [ ] Never true [ ] DK or Refused | 3 - HH3 |
| 4 | “(I/we) couldn’t afford to eat balanced meals.” Was that often, sometimes, or never true for (you/your household) in the last 12 months? [ ] Often true [ ] Sometimes true [ ] Never true [ ] DK or Refused |  | HH4 | “(I/we) couldn’t afford to eat balanced meals.” Was that often, sometimes, or never true for (you/your household) in the last 12 months? [ ] Often true [ ] Sometimes true [ ] Never true [ ] DK or Refused | 4-HH4 |
| 5 | “(I/we) relied on only a few kinds of low-cost food to feed (my/our) child/the children) because (I was/we were) running out of money to buy food.” Was that often, sometimes, or never true for (you/your household) in the last 12 months? [ ] Often true [ ] Sometimes true [ ] Never true [ ] DK or Refused |  | AD1 | In the last 12 months, since last (name of current month), did (you/you or other adults in your household) ever cut the size of your meals or skip meals because there wasn't enough money for food? [ ] Yes [ ] No (Skip AD1a) [ ] DK (Skip AD1a) | 5-CH1 |
| 6 | “(I/We) couldn’t feed (my/our) child/the children) a balanced meal, because (I/we) couldn’t afford that.” Was that often, sometimes, or never true for (you/your household) in the last 12 months? [ ] Often true [ ] Sometimes true [ ] Never true [ ] DK or Refused |  | AD1a | [IF YES ABOVE, ASK] How often did this happen—almost every month, some months but not every month, or in only 1 or 2 months? [ ] Almost every month [ ] Some months but not every month [ ] Only 1 or 2 months [ ] DK | 6-CH2 |
| 7 | "(My/Our child was/The children were) not eating enough because (I/we) just couldn't afford enough food." Was that often, sometimes, or never true for (you/your household) in the last 12 months? [ ] Often true [ ] Sometimes true [ ] Never true [ ] DK or Refused |  | AD2 | In the last 12 months, did you ever eat less than you felt you should because there wasn't enough money for food? [ ] Yes [ ] No [ ] DK | 7-CH3 |
| 8 | In the last 12 months, since last (name of current month), did (you/you or other adults in your household) ever cut the size of your meals or skip meals because there wasn't enough money for food? [ ] Yes [ ] No (SKIP 8a) [ ] DK or R (SKIP 8a) |  | AD3 | In the last 12 months, were you every hungry but didn't eat because there wasn't enough money for food? [ ] Yes [ ] No [ ] DK | 8-AD1 |
| 8a | [IF YES ABOVE, ASK] How often did this happen---almost every month, some months but not every month, or in only 1 or 2 months? [ ] Almost every month [ ] Some months but not every month [ ] Only 1 or 2 months [ ] DK |  | AD4 | In the last 12 months, did you lose weight because there wasn't enough money for food? [ ] Yes [ ] No [ ] DK | 8a-AD1a |
| 9 | In the last 12 months, did you ever eat less than you felt you should because there wasn't enough money to buy food? [ ] Yes [ ] No [ ] DK or R |  | AD5 | In the last 12 months, did (you/you or other adults in your household) ever not eat for a whole day because there wasn't enough money for food? [ ] Yes [ ] No (Skip AD5a) [ ] DK (Skip AD5a) | 9-AD2 |
| 10 | In the last 12 months, were you every hungry but didn't eat because you couldn't afford enough food? [ ] Yes [ ] No [ ] DK or R |  | AD5a | [IF YES ABOVE, ASK] How often did this happen—almost every month, some months but not every month, or in only 1 or 2 months? [ ] Almost every month [ ] Some months but not every month [ ] Only 1 or 2 months [ ] DK | 10-AD3 |
| 11 | In the last 12 months, did you lose weight because you didn't have enough money for food? [ ] Yes [ ] No [ ] DK or R |  |  | *Now I'm going to read you several statements that people have made about the food situation of their children. For these statements, please tell me whether the statement was OFTEN true, SOMETIMES true, or NEVER true in the last 12 months for (your child/children living in the household who are under 18 years old).* | 11-AD4 |
| 12 | In the last 12 months, did (you/you or other adults in your household) ever not eat for a whole day because there wasn't enough money for food? [ ] Yes [ ] No (SKIP 12a) [ ] DK or R (SKIP 12a) |  | CH1 | “(I/we) relied on only a few kinds of low-cost food to feed (my/our) child/the children) because (I was/we were) running out of money to buy food.” Was that often, sometimes, or never true for (you/your household) in the last 12 months? [ ] Often true [ ] Sometimes true [ ] Never true [ ] DK or Refused | 12-AD5 |
| 12a | [IF YES ABOVE, ASK] How often did this happen---almost every month, some months but not every month, or in only 1 or 2 months? [ ] Almost every month [ ] Some months but not every month [ ] Only 1 or 2 months [ ] DK or R |  | CH2 | “(I/We) couldn’t feed (my/our) child/the children) a balanced meal, because (I/we) couldn’t afford that.” Was that often, sometimes, or never true for (you/your household) in the last 12 months? [ ] Often true [ ] Sometimes true [ ] Never true [ ] DK or Refused | 12a-AD5a |
|  | *The next questions are about children living in the household who are under 18 years old.* |  | CH3 | "(My/Our child was/The children were) not eating enough because (I/we) just couldn't afford enough food." Was that often, sometimes, or never true for (you/your household) in the last 12 months? [ ] Often true [ ] Sometimes true [ ] Never true [ ] DK or Refused |  |
| 13 | In the last 12 months, since (current month) of last year, did you ever cut the size of (your child's/any of the children's) meals because there wasn't enough money for food? [ ] Yes [ ] No [ ] DK or R |  | CH4 | In the last 12 months, since (current month) of last year, did you ever cut the size of (your child's/any of the children's) meals because there wasn't enough money for food? [ ] Yes [ ] No [ ] DK | 13-CH4 |
| 14 | In the last 12 months, did (CHILD’S NAME/any of the children) ever skip meals because there wasn't enough money for food? [ ] Yes [ ] No (SKIP 14a) [ ] DK or R (SKIP 14a) |  | CH5 | In the last 12 months, did (CHILD’S NAME/any of the children) ever skip meals because there wasn't enough money for food? [ ] Yes [ ] No (Skip CH5a) [ ] DK (Skip CH5a) | 14-CH5 |
| 14a | [IF YES ABOVE ASK] How often did this happen---almost every month, some months but not every month, or in only 1 or 2 months? [ ] Almost every month [ ] Some months but not every month [ ] Only 1 or 2 months [ ] DK or R |  | CH5a | [IF YES ABOVE ASK] How often did this happen—almost every month, some months but not every month, or in only 1 or 2 months? [ ] Almost every month [ ] Some months but not every month [ ] Only 1 or 2 months [ ] DK | 14a-CH5a |
| 15 | In the last 12 months, (was your child/ were the children) ever hungry but you just couldn't afford more food? [ ] Yes [ ] No [ ] DK or R |  | CH6 | In the last 12 months, (was your child/were the children) ever hungry but you just couldn't afford more food? [ ] Yes [ ] No [ ] DK | 15-CH6 |
| 16 | In the last 12 months, did (your child/any of the children) ever not eat for a whole day because there wasn't enough money for food? [ ] Yes [ ] No [ ] DK or R |  | CH7 | In the last 12 months, did (your child/any of the children) ever not eat for a whole day because there wasn't enough money for food? [ ] Yes [ ] No [ ] DK | 16-CH7 |
|  | DK, Do not know; R, Refuse |  |  |  |  |

**Supplementary Table 2:** Item correlations of the 2000 and 2012 versions of the United States Household Food Security Survey Module

| **2000 version** | | | | | | | | | | | | | | | | | | | | | | | | | | | | |
| --- | --- | --- | --- | --- | --- | --- | --- | --- | --- | --- | --- | --- | --- | --- | --- | --- | --- | --- | --- | --- | --- | --- | --- | --- | --- | --- | --- | --- |
|  | | HH1 | | HH2 | | HH3 | | AD1 | | AD2 | | AD3 | | AD4 | | AD5 | | | CH1 | | CH2 | | CH3 | | CH4/CH5 | | CH6 | |
| HH1 | | 1 | |  | |  | |  | |  | |  | |  | |  | | |  | |  | |  | |  | |  | |
| HH2 | | 0.84 | | 1.00 | |  | |  | |  | |  | |  | |  | | |  | |  | |  | |  | |  | |
| HH3 | | 0.80 | | 0.83 | | 1.00 | |  | |  | |  | |  | |  | | |  | |  | |  | |  | |  | |
| AD1 | | 0.75 | | 0.79 | | 0.95 | | 1.00 | |  | |  | |  | |  | | |  | |  | |  | |  | |  | |
| AD2 | | 0.76 | | 0.85 | | 0.81 | | 0.91 | | 1.00 | |  | |  | |  | | |  | |  | |  | |  | |  | |
| AD3 | | 0.73 | | 0.88 | | 0.93 | | 0.89 | | 0.84 | | 1.00 | |  | |  | | |  | |  | |  | |  | |  | |
| AD4 | | 0.66 | | 0.72 | | 0.93 | | 0.97 | | 0.87 | | 0.89 | | 1.00 | |  | | |  | |  | |  | |  | |  | |
| AD5 | | 0.83 | | 0.85 | | 0.84 | | 0.88 | | 0.89 | | 0.83 | | 0.81 | | 1.00 | | |  | |  | |  | |  | |  | |
| CH1 | | 0.83 | | 0.83 | | 0.82 | | 0.78 | | 0.78 | | 0.90 | | 0.73 | | 0.84 | | | 1.00 | |  | |  | |  | |  | |
| CH2 | | 0.82 | | 0.84 | | 0.87 | | 0.77 | | 0.73 | | 0.91 | | 0.75 | | 0.85 | | | 0.95 | | 1.00 | |  | |  | |  | |
| CH3 | | 0.72 | | 0.77 | | 0.82 | | 0.83 | | 0.85 | | 0.91 | | 0.84 | | 0.90 | | | 0.93 | | 0.92 | | 1.00 | |  | |  | |
| CH4/CH5 | | 0.86 | | 0.86 | | 0.87 | | 0.77 | | 0.73 | | 0.87 | | 0.73 | | 0.68 | | | 0.86 | | 0.86 | | 0.73 | | 1.00 | |  | |
| CH6 | | 0.84 | | 0.68 | | 0.89 | | 0.82 | | 0.66 | | 0.83 | | 0.81 | | 0.77 | | | 0.88 | | 0.89 | | 0.83 | | 0.85 | | 1.00 | |
| **2012 version** | | | | | | | | | | | | | | | | | | | | | | | | | | | | |
|  | HH2 | | HH3 | | HH4 | | AD1 | | AD2 | | AD3 | | AD4 | | AD5 | | CH1 | CH2 | | CH3 | | CH4 | | CH5 | | CH6 | | CH7 |
| HH2 | 1.00 | |  | |  | |  | |  | |  | |  | |  | |  |  | |  | |  | |  | |  | |  |
| HH3 | 0.88 | | 1.00 | |  | |  | |  | |  | |  | |  | |  |  | |  | |  | |  | |  | |  |
| HH4 | 0.81 | | 0.88 | | 1.00 | |  | |  | |  | |  | |  | |  |  | |  | |  | |  | |  | |  |
| AD1 | 0.79 | | 0.84 | | 0.82 | | 1.00 | |  | |  | |  | |  | |  |  | |  | |  | |  | |  | |  |
| AD2 | 0.90 | | 0.82 | | 0.79 | | 0.92 | | 1.00 | |  | |  | |  | |  |  | |  | |  | |  | |  | |  |
| AD3 | 0.92 | | 0.86 | | 0.81 | | 0.87 | | 0.94 | | 1.00 | |  | |  | |  |  | |  | |  | |  | |  | |  |
| AD4 | 0.91 | | 0.85 | | 0.79 | | 0.91 | | 0.98 | | 0.96 | | 1.00 | |  | |  |  | |  | |  | |  | |  | |  |
| AD5 | 0.52 | | 0.51 | | 0.49 | | 0.53 | | 0.40 | | 0.42 | | 0.43 | | 1.00 | |  |  | |  | |  | |  | |  | |  |
| CH1 | 0.76 | | 0.84 | | 0.82 | | 0.83 | | 0.79 | | 0.79 | | 0.81 | | 0.50 | | 1.00 |  | |  | |  | |  | |  | |  |
| CH2 | 0.81 | | 0.86 | | 0.89 | | 0.86 | | 0.82 | | 0.82 | | 0.86 | | 0.52 | | 0.90 | 1.00 | |  | |  | |  | |  | |  |
| CH3 | 0.90 | | 0.88 | | 0.86 | | 0.82 | | 0.81 | | 0.86 | | 0.82 | | 0.52 | | 0.88 | 0.91 | | 1.00 | |  | |  | |  | |  |
| CH4 | 0.74 | | 0.81 | | 0.72 | | 0.84 | | 0.81 | | 0.85 | | 0.85 | | 0.38 | | 0.95 | 0.88 | | 0.86 | | 1.00 | |  | |  | |  |
| CH5 | 0.66 | | 0.71 | | 0.70 | | 0.58 | | 0.68 | | 0.71 | | 0.69 | | -0.13 | | 0.71 | 0.72 | | 0.73 | | 0.75 | | 1.00 | |  | |  |
| CH6 | 0.69 | | 0.71 | | 0.70 | | 0.72 | | 0.81 | | 0.79 | | 0.80 | | -0.11 | | 0.70 | 0.72 | | 0.71 | | 0.77 | | 0.95 | | 1.00 | |  |
| CH7 | 0.68 | | 0.71 | | 0.69 | | 0.72 | | 0.81 | | 0.79 | | 0.79 | | -0.12 | | 0.69 | 0.72 | | 0.71 | | 0.77 | | 0.95 | | 1.00 | | 1.00 |

| **Supplementary Table 3:** Item fit Chi-square of the 2000 and 2012 versions of the United States Household Food  Security Survey Module | | | |
| --- | --- | --- | --- |
|  | ***p*-value** | | |
| **Items*** | **2000 version** | **2012 version** | |
|  | *All items* | *Household/adults* | *Children* |
| *“(I/We) worried whether (my/our) food would run out before (I/we) got money to buy more.” Was that often true, sometimes true, or never true for (you/your household) in the last 12 months?* | 0.475 | 0.436 |  |
| *“The food that (I/we) bought just didn’t last, and (I/we) didn’t have money to get more.” Was that often, sometimes, or never true for (you/your household) in the last 12 months?* | 0.100 | 0.139 |  |
| *“(I/we) couldn’t afford to eat balanced meals.” Was that often, sometimes, or never true for (you/your household) in the last 12 months?* | 0.406 | 0.545 |  |
| *In the last 12 months, since last (name of current month), did (you/you or other adults in your household) ever cut the size of your meals or skip meals because there wasn't enough money for food?* | 0.238 | 0.129 |  |
| *In the last 12 months, did you ever eat less than you felt you should because there wasn't enough money for food?* | 0.426 | 0.713 |  |
| *In the last 12 months, were you every hungry but didn't eat because there wasn't enough money for food?* | 0.178 | 0.921 |  |
| *In the last 12 months, did you lose weight because there wasn't enough money for food?* | 0.188 | 0.842 |  |
| *In the last 12 months, did (you/you or other adults in your household) ever not eat for a whole day because there wasn't enough money for food?* | 0.386 | 0.683 |  |
| *“(I/we) relied on only a few kinds of low-cost food to feed (my/our) child/the children) because (I was/we were) running out of money to buy food.” Was that often, sometimes, or never true for (you/your household) in the last 12 months?* | 0.545 |  | 0.426 |
| *“(I/We) couldn’t feed (my/our) child/the children) a balanced meal, because (I/we) couldn’t afford that.” Was that often, sometimes, or never true for (you/your household) in the last 12 months?* | 0.614 |  | 0.188 |
| *"(My/Our child was/The children were) not eating enough because (I/we) just couldn't afford enough food." Was that often, sometimes, or never true for (you/your household) in the last 12 months?* | 0.297 |  | 0.297 |
| *In the last 12 months, since (current month) of last year, did you ever cut the size of (your child's/any of the children's) meals because there wasn't enough money for food?* | 0.465 |  | 0.139 |
| *In the last 12 months, did (CHILD’S NAME/any of the children) ever skip meals because there wasn't enough money for food?* |  |  | 1.000 |
| *In the last 12 months, (was your child/were the children) ever hungry but you just couldn't afford more food?* | 0.426 |  | 0.713 |
| *In the last 12 months, did (your child/any of the children) ever not eat for a whole day because there wasn't enough money for food?* |  |  | 0.535 |
| * Items wording of the 2012 version of the United States Household Food Security Survey Module | | | |

| **Supplementary Table 4:** 2-Parameter logistic latent trait models of the 2000 and 2012 versions of the United States Household Food Security Survey Module | | | | | | | | | | | |
| --- | --- | --- | --- | --- | --- | --- | --- | --- | --- | --- | --- |
|  | **2000** | | |  | **2012** | | | | | | |
| **Items*** | **Difficulty** | **Discrimination** | **Standardized**  **factor loadings** |  | **Difficulty** | | **Discrimination** | | **Standardized**  **factor loadings** | |  |
|  |  |  |  |  | **Household and Adults** | **Children** | **Household and Adults** | **Children** | **Household and Adults** | **Children** |  |
| *“(I/We) worried whether (my/our) food would run out before (I/we) got money to buy more.” Was that often true, sometimes true, or never true for (you/your household) in the last 12 months?* | 1.04 | 3.37 | 0.96 |  | 0.95 |  | 4.98 |  | 0.98 |  |  |
| *“The food that (I/we) bought just didn’t last, and (I/we) didn’t have money to get more.” Was that often, sometimes, or never true for (you/your household) in the last 12 months?* | 1.29 | 4.29 | 0.97 |  | 1.65 |  | 4.77 |  | 0.98 |  |  |
| *“(I/we) couldn’t afford to eat balanced meals.” Was that often, sometimes, or never true for (you/your household) in the last 12 months?* | 1.11 | 3.97 | 0.97 |  | 1.93 |  | 3.33 |  | 0.96 |  |  |
| *In the last 12 months, since last (name of current month), did (you/you or other adults in your household) ever cut the size of your meals or skip meals because there wasn't enough money for food?* | 2.05 | 4.98 | 0.98 |  | 2.35 |  | 4.70 |  | 0.98 |  |  |
| *In the last 12 months, did you ever eat less than you felt you should because there wasn't enough money for food?* | 1.94 | 4.33 | 0.97 |  | 2.22 |  | 7.52 |  | 0.99 |  |  |
| *In the last 12 months, were you every hungry but didn't eat because there wasn't enough money for food?* | 2.07 | 22.05 | 1.00 |  | 2.65 |  | 6.11 |  | 0.99 |  |  |
| *In the last 12 months, did you lose weight because there wasn't enough money for food?* | 2.35 | 5.47 | 0.98 |  | 2.72 |  | 18.97 |  | 1.00 |  |  |
| *In the last 12 months, did (you/you or other adults in your household) ever not eat for a whole day because there wasn't enough money for food?* | 2.72 | 4.93 | 0.98 |  | 3.93 |  | 2.95 |  | 0.95 |  |  |
| *“(I/we) relied on only a few kinds of low-cost food to feed (my/our) child/the children) because (I was/we were) running out of money to buy food.” Was that often, sometimes, or never true for (you/your household) in the last 12 months?* | 1.13 | 5.23 | 0.98 |  |  | 1.47 |  | 4.18 |  | 0.97 |  |
| *“(I/We) couldn’t feed (my/our) child/the children) a balanced meal, because (I/we) couldn’t afford that.” Was that often, sometimes, or never true for (you/your household) in the last 12 months?* | 1.31 | 20.42 | 1.00 |  |  | 2.04 |  | 9.42 |  | 0.99 |  |
| *"(My/Our child was/The children were) not eating enough because (I/we) just couldn't afford enough food." Was that often, sometimes, or never true for (you/your household) in the last 12 months?* | 2.02 | 6.49 | 0.99 |  |  | 2.27 |  | 9.58 |  | 0.99 |  |
| *In the last 12 months, since (current month) of last year, did you ever cut the size of (your child's/any of the children's) meals because there wasn't enough money for food?* | 2.57 | 4.08 | 0.97 |  |  | 2.68 |  | 4.66 |  | 0.98 |  |
| *In the last 12 months, did (CHILD’S NAME/any of the children) ever skip meals because there wasn't enough money for food?* |  |  |  |  |  | 2.82 |  | 39.21 |  | 1.00 |  |
| *In the last 12 months, (was your child/were the children) ever hungry but you just couldn't afford more food?* | 2.55 | 5.11 | 0.98 |  |  | 3.32 |  | 49.06 |  | 1.00 |  |
| *In the last 12 months, did (your child/any of the children) ever not eat for a whole day because there wasn't enough money for food?* | - | - | - |  |  | 3.49 |  | 35.60 |  | 1.00 |  |
| * Items’ wording of the 2012 version of the United States Household Food Security Survey Module | | | | | | | | | | | |
